# Pavlovian to Instrumental Transfer Responses Do Not Correlate With Addiction-Like Behavior in Rats

**DOI:** 10.3389/fnbeh.2019.00129

**Published:** 2019-06-18

**Authors:** Tatiane T. Takahashi, Valentina Vengeliene, Thomas Enkel, Sara Reithofer, Rainer Spanagel

**Affiliations:** ^1^Institute of Psychopharmacology, Central Institute of Mental Health (CIMH), Heidelberg University, Faculty of Medicine Mannheim, Mannheim, Germany; ^2^Faculty of Medicine Mannheim, Department of Molecular Biology, Central Institute of Mental Health, Heidelberg University, Mannheim, Germany

**Keywords:** cocaine self-administration, 0/3-criteria rat model of cocaine addiction, outcome-specific PIT, general PIT, relapse

## Abstract

Pavlovian learning plays a prominent role in the etiology of addiction. The influence of Pavlovian conditioning on the expression of an instrumental response can be studied using the Pavlovian-to-instrumental transfer (PIT) paradigm. This paradigm consists of independent Pavlovian conditioning and instrumental training prior to the combination of both during the test. During this test, the reward is not available, and an increase in the instrumental responding during conditioned stimuli presentation is a measure of PIT. Recent studies have reported a higher PIT in alcohol and nicotine dependent patients, suggesting that enhanced PIT might be a marker for dependence vulnerability. However, these studies did not use standard PIT procedures, and a clear correlation between an enhanced PIT and drug-related and addictive behaviors has so far not been demonstrated. For a systematic evaluation rats were trained in a cocaine addiction model. Addicted-like and non-addicted-like rats were subsequently assessed in the PIT paradigm. In a further experiment, rats were first tested in the PIT paradigm and thereafter subjected to cocaine self-administration (CSA) training. Our results revealed that addicted-like rats did not differ from non-addicted-like in their performance in the PIT test. However, CSA behavior showed a positive correlation with PIT. This data suggests that stronger PIT may predict higher motivational impact of conditioned stimuli on drug self-administration and improved learning of drug-cue association rather than the risk to develop addiction as such.

## Introduction

Addiction theories postulate that Pavlovian learning plays a key role in the development of drug addiction and maintenance of drug use. Pavlovian learning involves transfer of the motivational value of the primary reward to the conditioned stimulus (CS) associated with drug availability during the course of drug use (Berridge and Robinson, 2003; Sanchis-Segura and Spanagel, 2006). Such a CS can impact on ongoing instrumental behavior, even if the instrumental behavior is acquired independently of Pavlovian conditioning. This process is called Pavlovian-to-Instrumental transfer (PIT; Colwill and Rescorla, 1988; Holmes et al., 2010; Cartoni et al., 2013, 2016). In PIT, positively valued Pavlovian cues promote instrumental responses and approach (e.g., enhance the frequency of pressing a button in order to obtain a drug; Cartoni et al., 2016). Pavlovian conditioned cues can thus bias instrumental behavior towards drug seeking and intake in both drug abusers and animals trained to self-administer drugs (Everitt et al., 2001; Glasner et al., 2005; Weiss, 2005; Hogarth et al., 2007; LeBlanc et al., 2012). Although it is well established that drug conditioned cues play a critical role in drug addiction the role of PIT on drug-related and addictive behaviors is less clear (Hogarth et al., 2018).

In the PIT paradigm, subjects are first trained in Pavlovian stimulus-outcome sessions, separately from the instrumental response-outcome sessions, to prevent development of the association between the CS and instrumental action. During the PIT test, presentation of the CS increases the instrumental response rate, demonstrating the “energizing” properties of the CS on the expression of the instrumental behavior. In outcome-specific PIT, presenting a particular reward-predicting cue can selectively elevate instrumental responses that are associated with the same unique reward, while in general PIT, a reward predicting cue can generally modify instrumental responses towards any outcome (Holmes et al., 2010; Cartoni et al., 2016).

PIT have been widely studied in animals, also in the context of drugs of abuse, demonstrating that drug-experienced (cocaine and alcohol) animals exhibit an enhanced PIT (Corbit and Janak, 2007; Holmes et al., 2010; Cartoni et al., 2016; Lamb et al., 2016). However, it has also been suggested that specific PIT effects are abolished following drug exposure (Shiflett, 2012; Hogarth et al., 2013). Studies in young adult smokers (Hogarth and Chase, 2011, 2012; Hogarth, 2012; Hogarth et al., 2015), young adult drinkers (Martinovic et al., 2014; Hardy et al., 2017) and treatment-engaged addicts (Hogarth et al., 2018) did not demonstrate a link between specific PIT effects and dependence or dependence severity. However, Garbusow et al. (2014) and Schad et al. (2019) showed that in alcohol-dependent patients both alcohol-related as well as non-alcohol-related PIT occurred more frequently than in healthy controls. Therefore, it is unclear whether general PIT might be different in addicts, and if this could be used as a marker of addictive behavior.

Here, we used a general PIT model for natural rewards and studied two research questions in a rat model of cocaine self-administration (CSA) and cocaine addiction: (i) given that cocaine-experienced rats may exhibit an enhanced PIT effect (Lamb et al., 2016) we asked if the performance in CSA correlates with the strength of PIT; and (ii) provided that general PIT seems to occur more frequently in addicted patients we asked if an enhanced PIT occurs in cocaine-addicted compared to non-addicted rats.

To evaluate if behavior during the PIT test correlates with addiction-like features, the DSM-based 0/3 criteria animal model of cocaine addiction was used. This animal model has a good face and construct validity (Deroche-Gamonet et al., 2004; Cannella et al., 2013, 2018; Spanagel, 2017). The model is based on long-term CSA that produces a ratio of cocaine addicted-like rats, equivalent to the addicted population of human cocaine users (Anthony et al., 1994). Following the establishment of an appropriate PIT paradigm (adapted from Holland, 2004) two experiments were performed: (i) rats were trained in the cocaine addiction model, and subsequently addicted-like and non-addicted-like rats were assessed in the PIT paradigm (ii) rats were first tested in the PIT paradigm and thereafter subjected to CSA training.

## Materials and Methods

### Animals

Thirty-two-month-old male Wistar rats from Harlan Laboratories (Derby, United Kingdom) were used for the establishment of the PIT protocol. Other 68 2-month-old male Sprague-Dawley rats from Charles River Laboratories (Sulzfeld, Germany) was used for CSA and cocaine addiction model and PIT testing. All animals were acclimatized in the laboratory facilities for a week before catheter implantation or the initiation of behavioral experiments. Animals were housed individually in standard rat cages (Type-III; Ehret, Emmendingen, Germany) throughout the study, and maintained under reverse light/dark cycle (lights on at 5:00, lights off at 17:00). Temperature was controlled (22 ± 2°C), drinking water was provided *ad libitum* unless indicated otherwise. Twenty-grams of standard laboratory rat food (Sniff, Soest, Germany) was given daily for the rat group used in the cocaine addiction model, and rats used for the establishment of the PIT protocol were fed *ad libitum*. All experimental procedures were approved by the Committee on Animal Care and Use (Regierungspräsidium Karlsruhe) and carried out in accordance with the local Animal Welfare Act and the European Communities Council Directive of 22 September 2010 (2010/63/EU).

### Drugs

Cocaine hydrochloride (Sigma-Aldrich, Taufkirchen, Germany) was dissolved in sterile saline.

### Experimental Design

Two groups of rats were used to establish a PIT paradigm: a group that received both Pavlovian conditioning and instrumental training (PIT group, *n* = 24), and a control group (*n* = 8) that was prevented from stimulus-reward pairings during Pavlovian conditioning but had identical instrumental training. The control group was included to measure any unconditioned effects of stimulus presentation on responding.

For Experiment 1, another group of rats was trained in the cocaine addiction model (*n* = 48). Cocaine addicted-like (3 criteria group, *n* = 7) and non-addicted-like (0 criteria group, *n* = 8) were subsequently assessed in the PIT paradigm.

To assess whether training of rats in the addiction model had no carryover effect on PIT performance, Experiment 2 employed a new group of naïve rats (*n* = 20), which were subjected first to the PIT paradigm and thereafter to CSA training.

### PIT Paradigm

The training protocol used in the present study was adapted from Holland (2004) and followed several steps: habituation, Pavlovian conditioning, instrumental training and Pavlovian reconditioning. PIT test was done next after the last training session.

### Operant Self-administration Apparatus for Pavlovian Instrumental Transfer

PIT paradigm was carried out in operant chambers (MED Associates Inc., St. Albans, VT, USA) enclosed in ventilated sound-attenuating cubicles. The chambers were equipped with two levers placed at opposite walls. Responses at the active lever activated a syringe pump that delivered 60 μl of 10% (w/v) sucrose solution into a liquid receptacle, which was placed on the left side of the lever. Responses at the inactive lever were recorded but had no programmed consequences. A cue-light stimulus was attached above both response levers of the operant chamber. Delivery of sucrose, presentation of CS and data recording were controlled by a computer with MED-PC software (MED Associates).

#### Magazine Training

Before the magazine training session, animals were water deprived for approximately 22 h to increase exploratory and consummatory behavior. Magazine training lasted for 1 h, and during this time sucrose was delivered at varying and unpredictable time intervals of 2 min on average (variable-interval, VI) but neither levers nor cue-light was presented.

#### Pavlovian Conditioning

Pavlovian conditioning was done in 60 min daily sessions for five consecutive days and levers were withdrawn throughout these sessions. Animals were not water deprived during this phase. During the conditioning session, a constant-cue-light, situated above the active lever, was presented for 2 min at random time intervals (inter-trial interval was 2–3 min) and served as the CS. Presentation of the CS was accompanied with an immediate delivery of 4–5 sucrose reinforcers [one reward corresponded to 60 μl of 10% (w/v) sucrose solution delivered into a liquid receptacle]. A total of nine pairings of CS and reward were given per session. The control group received the corresponding amount of sucrose (~2.5 ml) in the receptacle at the beginning of the session to avoid association between the CS and reward.

#### Instrumental Training

Instrumental training started on the next day following Pavlovian conditioning. Prior to the instrumental training, all animals were water deprived for 22 h to promote acquisition of lever responding. In this phase, cue-lights were switched-off and sucrose reinforcers were granted by pressing the active lever. Each daily session lasted 30 min and VI was increased progressively across the training days. In the first 3 days, animals were trained on a FR1 schedule of reinforcement for acquisition of instrumental response. On the subsequent 10 days, the average VI changed as follows: a training day on VI-10 (5 s, 10 s, 15 s), followed by a day on VI-20 (10 s, 20 s, 30 s) and finalizing with eight training days on VI-30 (10 s, 20 s, 30 s, 40 s, 50 s). Both active and inactive levers were available throughout the instrumental training; however, responses at the inactive lever did not result in reward delivery.

#### Pavlovian Reconditioning

All animals were subjected to a single conditioning session of 30 min the day prior to the PIT test. The conditions were the same as during the Pavlovian conditioning sessions described above.

#### Pavlovian Instrumental Transfer Test

Both active and inactive levers were available throughout the PIT test. CS was presented randomly four times for 2 min in a single 36-min session, and the intertrial interval was 4–10 min. A period of 2-min duration just before CS presentation was considered as the pre-CS period. Responding on both active and inactive levers was recorded throughout the PIT test and the number of active lever-presses prior (pre-CS) and during CS presentation was used to demonstrate the transfer effect. Neither active nor inactive lever-responding was reinforced by sucrose delivery.

### Experiment 1: PIT in Cocaine Addicted-Like and Non-addicted-Like Rats

#### Surgery

A polyurethane catheter (internal diameter: 0.58 mm, external diameter: 0.94 mm) was implanted in the jugular vein under isoflurane anesthesia (induction: 5%; maintenance: ~2.5%). The proximal end of the catheter was placed in the right atrium of the animal’s heart, while the distal end was fixed in the mid scapular region. Rats were allowed to recover for 4–7 days after the surgery. Catheters were flushed daily with unfractionated heparin (100 IU/ml) solution containing enrofloxacin (Baytril^®^, 1 mg/ml).

#### Operant Cocaine Self-administration Apparatus

CSA trainings were carried out in nose-poke operant chambers (40 cm long × 30 cm width × 52 cm high; Imetronic, France) enclosed in ventilated sound-attenuating cubicles. Two nose-poke holes at opposite walls, 5 cm above the grid floor recorded the responses by the interruption of a photo-beam projected across the hole. Poking in the active hole resulted in the delivery of an infusion of 0.8 mg/kg of cocaine, whereas poking in the inactive hole had no programmed consequences. The chambers were also equipped with a white cue-light placed 9.5 cm above the grid floor, a green cue-light next to it, a blue cue-light located on the opposite wall 33 cm above the grid floor and a house light that illuminated the entire chamber. Data was collected using POLY software.

#### Cocaine Self-administration Training

CSA protocol was performed as initially described by Deroche-Gamonet et al. (2004) and in our previous work (Cannella et al., 2013, 2018; Vengeliene et al., 2018). Briefly, each CSA session consisted of alternated periods of drug availability (drug-ON, 40 min) and non-availability (NO-drug, 15 min). During drug-ON periods, a blue cue light signaled the availability of cocaine at FR5 schedule of reinforcement. If the schedule was completed within 40 s time, an infusion of cocaine (0.8 mg/kg/infusion) was delivered paired with presentation of a white cue-light. During NO-drug periods, blue and white cue-lights were withdrawn and a house light indicated non-availability of cocaine. Nose-pokes had no scheduled consequences but recorded during NO-drug periods. Each CSA session lasted 2.5 h or session was ceased after 35 cocaine infusions a day.

Following 45 CSA training sessions, three addiction criteria were tested: (1) motivation to self-administer cocaine; (2) persistence of cocaine-seeking; and (3) resistance to punishment.

#### Motivation to Self-administer the Drug

Breakpoint test was used to assess animals’ motivation to take cocaine. Test was based on the progressive-ratio schedule of reinforcement. Drug availability was signaled by the blue cue light and the ratio of responses was increased after each infusion according to the following progression: 10, 20, 30, 45, 65, 85, 115, 145, 185, 225, 275, 325, 385, 445, 515, 585, 665, 745, 835, 925, 1,025, 1,125, 1,235, 1,345, 1,465, and 1,585. The last completed ratio performed by the rat was used as a measurement of animals’ motivation. The test elapsed either after 6 h or when the ratio was not completed within 1 h time.

#### Persistence of Cocaine-Seeking

Persistence of cocaine-seeking was measured as the number of active nose-pokes during the NO-drug periods averaged in the last four CSA training sessions prior to the BP test.

#### Resistance to Punishment

Test consisted of cocaine infusion paired with mild foot-shocks (0.2 mA, 1 s). Additionally to the blue and white cue lights, a green cue-light was lit immediately after the first active nose-poke to indicate the presence of shock. FR5 was used as the schedule of reinforcement; however, a foot-shock was delivered at the completion of the fourth as well as the fifth active nose-poke, which was then paired with a cocaine infusion (0.8 mg/kg/infusion). Percentage of cocaine infusions earned during the 40 min test in relation to the baseline infusions during the first drug-ON period in the last four CSA training sessions prior to the test was used as the measurement of resistance to punishment.

#### Addiction Criteria Classification

For each addiction-like behavioral criterion, a score of 0 or 1 was given to the animals depending on their performance. According to our previous studies (Cannella et al., 2018; Vengeliene et al., 2018) rats responding above 60th percentile of the population distribution received a score of 1, whereas animals responding below this threshold received a score of 0. The sum of scores from the three behavioral tests resulted in animals displaying 0–3 positive criteria. Rats positive for all three criteria were considered as addicted-like animals (3 criteria) rats, whereas animals negative for all criteria were considered as non-addicted-like (0 criteria) rats.

#### PIT Testing

Following characterization of addiction-like behaviors, 0 criteria and 3 criteria rats were subjected to further baseline CSA training, completing 55 CSA sessions. Thereafter, animals were left undisturbed for 1–2 weeks, and subsequently trained in the PIT paradigm as described above.

### Experiment 2: Cocaine Self-administration in PIT Tested Rats

After testing an additional group of naïve rats in the PIT paradigm, all rats underwent catheter implantation surgery as described above. Following the recovery, 20 rats were subjected to 33 CSA training sessions under the same conditions as in the Experiment 1, which was enough to establish stable responding.

### Data Analysis

Animal performance during the PIT test was analyzed using either a three-way analysis of variance (ANOVA) with repeated measures [factors were: condition (pre-CS vs. CS), group (either control vs. PIT or 0 criteria vs. 3 criteria) and lever (active vs. inactive)] or two-way ANOVA with repeated measures in case of a rat group used for CSA (factors were: condition and lever). For analysis of responses during the instrumental training in the PIT paradigm and CSA in 0 criteria vs. 3 criteria rats, a three-way ANOVA with repeated measures was used, and the factors were lever (active vs. inactive), group (0 criteria vs. 3 criteria) and training sessions. Reinforcers earned during instrumental training in the PIT paradigm and CSA was analyzed by two-way ANOVA with repeated measures [factors: group (0 criteria vs. 3 criteria) and training sessions]. Whenever significant differences were found, Newman-Keuls *post hoc* test was applied. Student *t*-test was used for the analysis of 0 criteria and 3 criteria rats in each addiction-like behavioral test. Pearson Correlation was used to assess linear relationship between performances in the CSA, addiction criteria and the PIT test. Performance during the last CSA sessions was calculated as [(number of active nose-pokes/number of total nose pokes)*100], and it demonstrated how well the animal learned to discriminate between reinforced (active) and non-reinforced (inactive) responding, and Performance in the PIT test was calculated as [number of active lever presses during CS presentations/(total number of active lever presses during both CS and pre-CS periods)*100], and it demonstrated whether the animal discriminates between CS and pre-CS periods. The chosen level of significance was *p* < 0.05.

## Results

### PIT Paradigm

**Figure 1:**
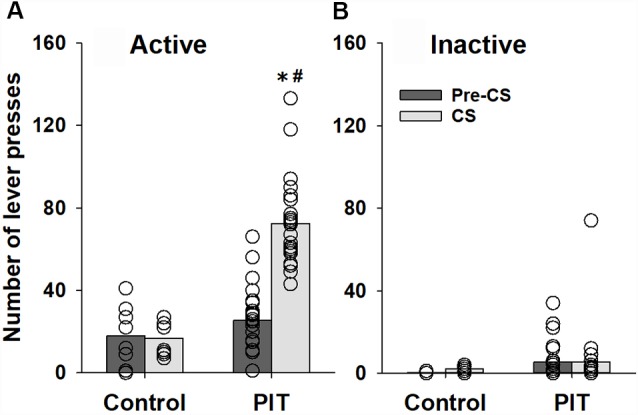
Establishing a Pavlovian instrumental transfer (PIT) test. Performance during the PIT test by control rats (*n* = 8) that were prevented from learning stimulus-reward association during Pavlovian conditioning sessions and rats that were subjected to five Pavlovian conditioning sessions prior to instrumental training (PIT, *n* = 24). The total number of **(A)** active and **(B)** inactive lever presses during four random 2-min conditioned stimulus (CS) presentations and during the 2-min periods just before CS presentations (pre-CS) are shown. Data are presented as means (bars) and individual values (open circles, please note that identical values are overlapping). *Indicates significant difference from pre-CS and ^#^indicates significant difference from the control group, *p* < 0.05.

Our results show that animals used for the establishment of the PIT paradigm increased lever responding during the CS presentations of the PIT test (factor condition: *F*_(1,120)_ = 16.2, *p* < 0.001) compared to the pre-CS condition. Three-way ANOVA revealed that lever-responding was different during the CS presentation between animals which received Pavlovian conditioning sessions (PIT group) and animals that were prevented from learning stimulus-reward association during Pavlovian conditioning sessions (control group; factor group: *F*_(1,120)_ = 37.6, *p* < 0.001, group × condition interaction: *F*_(1,120)_ = 15.5, *p* < 0.001 and group × condition × lever interaction: *F*_(1, 120)_ = 18.0, *p* < 0.001). The subsequent *post hoc* analysis demonstrated that these differences were caused by changes in responding on the active lever (Figure 1A). CS presentation had no significant effect on inactive lever-responding (Figure 1B).

### Characterization of Addiction-Like Behavior in the 0/3 Criteria Model

**Figure 2:**
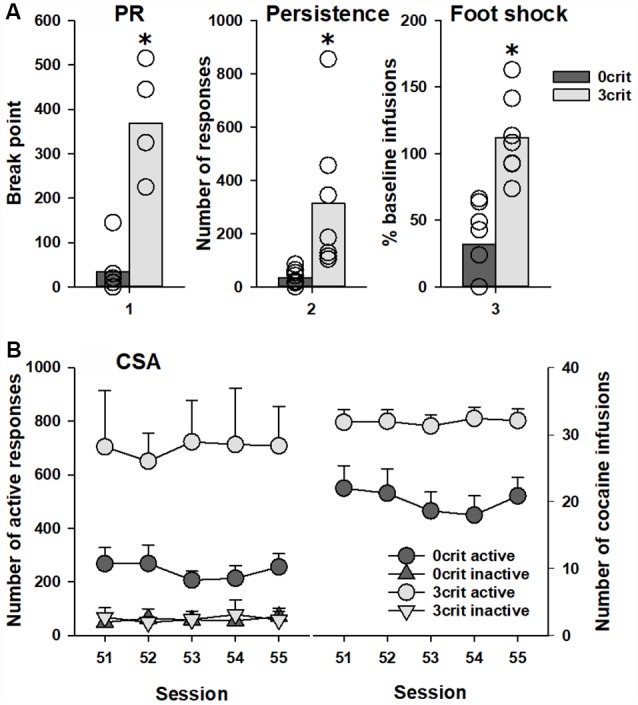
Behavioral characterization of 0 criteria and 3 criteria rats. Rats characterized by addicted-like phenotype (3 crit; *n* = 7) showed higher response than non-addicted-like rats (0 crit; *n* = 8) in each criterion for addiction-like behavior **(A)**: (1) motivation to self-administer cocaine measured in the breakpoint test as the last completed ratio in the progressive ratio schedule (PR); (2) persistence of drug-seeking measured as the number of active nose-pokes during the no-drug periods; and (3) resistance to punishment measured as the number of cocaine infusions earned in association with a foot-shock delivery in relation to the last four cocaine self-administration (CSA) training sessions prior to the test (%). Panel **(B)** shows the last five CSA sessions (51–55) as (left) number of responses in both the active and inactive hole and (right) the number of cocaine reinforcers earned during these sessions. Data are presented as **(A)** means (bars) and individual values (open circles, please note that identical values are overlapping) and **(B)** means ± SEM. Groups were different in number of both active responses and cocaine reinforcers, *Indicates significant difference from the 0 criteria group, *p* < 0.05.

Out of 48 rats that initiated the CSA training, 36 rats successfully completed it. This reduction in animal numbers occurred due to either loss of catheter potency or death of animals during training. A total of seven rats were characterized as positive for all addiction-like criteria (3 criteria), and classified as addicted-like rats, and 11 rats were negative for all criteria (0 criteria), and classified as non-addicted-like rats, eight of which were tested in the PIT paradigm. Student *t*-test showed significant difference between 0 criteria and 3 criteria rats in each of the addiction-like behaviors/criteria: motivation to take cocaine (*t*_(13)_ = 7.0, *p* < 0.001), persistence of drug-seeking (*t*_(13)_ = 2.8, *p* < 0.05) and resistance to punishment (*t*_(13)_ = 5.3, *p* < 0.001; Figure 2A). Analysis of data during the last CSA sessions demonstrated that number of active nose-pokes as well as number of cocaine infusions was significantly lower in 0 criteria rats compared to 3 criteria animals (factor group: *F*_(1,26)_ = 9.0, *p* < 0.01 and *F*_(1,13)_ = 11.8, *p* < 0.01 for the number of pokes and the number of cocaine reinforcers, respectively; Figure 2B). In conclusion, 0 criteria and 3 criteria rats shared identical experimental conditions but differed pronouncedly in their addictive-like behavior and could therefore be ideally used for further PIT testing.

### No Difference in PIT Testing in Cocaine Addicted-Like and Non-addicted-Like Rats

**Figure 3:**
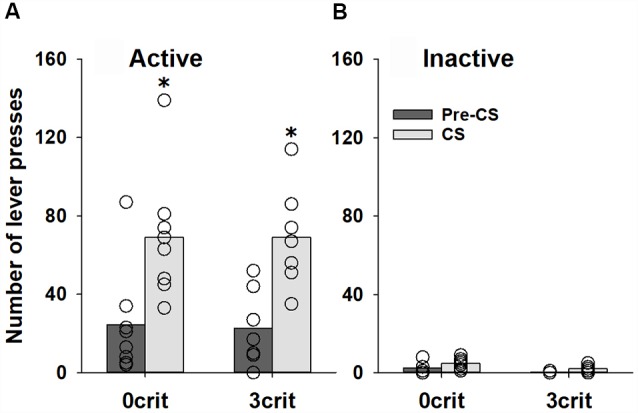
No difference in PIT between cocaine addicted and non-addicted rats. Performance during the PIT test in non-addicted-like (0 crit, *n* = 8) and addicted-like (3 crit, *n* = 7) rats subjected to five Pavlovian conditioning sessions prior to instrumental training. The total number of **(A)** active and **(B)** inactive lever presses during four random 2-min CS presentations and during the 2-min periods just before CS presentations (pre-CS) are shown. Data are presented as means (bars) and individual values (open circles, please note that identical values are overlapping). *Indicates significant difference from pre-CS, *p* < 0.05.

Analysis of the data obtained during the PIT training procedure demonstrated that behavior of 0 criteria and 3 criteria rats during the instrumental training did not differ (*p* = 0.21 and *p* = 0.63 for the number of responses and reinforcers earned, respectively), indicating that 0 criteria and 3 criteria rats received equivalent number of sucrose reinforcers during the instrumental training (data not shown). During the transfer test, the number of active lever presses increased during CS presentation in both (0 criteria and 3 criteria groups; factor condition: *F*_(1,52)_ = 23.0, *p* < 0.001). However, no difference was found between two groups (factor group: *p* = 0.74, group × condition interaction: *p* = 0.96 and group × condition × lever interaction: *p* = 0.91; Figure 3A). Responding on the inactive lever was not significantly affected by CS presentation (Figure 3B).

**Figure 4:**
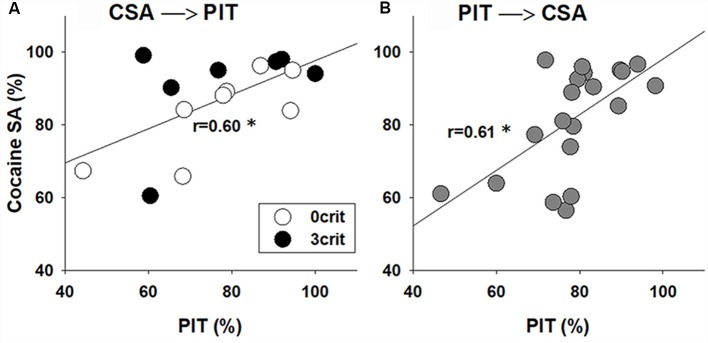
PIT correlates with the performance in CSA. Correlation analysis between performance during the last CSA sessions and the PIT test in rats that were first subjected to **(A)** 55 sessions of CSA and addiction-like behavioral testing that yielded addicted-like (3 crit) and non-addicted-like (0 crit) rats and then to PIT testing or **(B)** first were tested in the PIT paradigm and then subjected to CSA training. Performance during CSA demonstrates (Y-axis) how well the animal discriminate between reinforced (active) and non-reinforced (inactive) responding, and it was calculated as [(number of active nose-pokes/number of total nose pokes)*100]. Performance in the PIT test (X-axis) was calculated as [number of active lever presses during CS presentations/(total number of active lever presses during both CS and pre-CS periods)*100], and it demonstrates whether the animal discriminates between CS and pre-CS periods. *Indicates a significant correlation between performance in PIT and CSA, *p* < 0.05.

Given that cocaine-experienced rats exhibit an enhanced PIT (Lamb et al., 2016) we asked if the performance in CSA in the 0/3 criteria model correlates with the strength of PIT. Our analysis shows a positive correlation between rat performance during the last 5 CSA sessions (i.e., ability of a rat to discriminate between the active and inactive nose-hole, %) and the transfer in the Pavlovian to instrumental response (*r* = 0.60, *p* < 0.05; Figure 4A).

### PIT Correlates With the Performance in Cocaine Self-administration

To confirm the robustness of the correlation analysis and to ensure that there were no carryover effects from the prior experience with cocaine operant conditioning on PIT performance, a group of rats (*n* = 20) was subjected to a reversed experimental order. The rats were initially trained in the PIT paradigm and thereafter they were subjected to the cocaine-conditioned self-administration procedure. Similar to the previous experiment, there was a positive correlation between performance in the Pavlovian to instrumental response and the last five CSA training sessions (*r* = 0.61, *p* < 0.01; Figure 4B), demonstrating that the behavior during PIT may predict cocaine-taking behavior.

## Discussion

In the present study, rats subjected to Pavlovian stimulus-outcome conditioning increased instrumental lever responding upon CS presentation, which indicates the occurrence of a transfer effect. On the contrary, rats that did not receive CS pairing with the reward delivery during the Pavlovian conditioning phase did not show a transfer effect during the test. Addicted-like rats did not differ from non-addicted-like in their performance in the PIT test. However, there was a positive correlation between the performance in CSA behavior and PIT. Thus, animals that showed a high accuracy and preference for the cocaine reinforced responding also showed a higher PIT than animals that performed less well in the self-administration paradigm. The latter finding was confirmed in a reversed experimental approach where PIT was first assessed in cocaine-naïve rats; then rats were trained in the CSA paradigm, and after stable responding, their self-administration performance was measured. Again, a positive correlation between the strength of PIT and self-administration behavior was found. This is in line with previous PIT experiments in rats showing that drug-experienced animals exhibit an enhanced PIT (Holmes et al., 2010; Cartoni et al., 2016; Lamb et al., 2016) and extend this conclusion by the finding that an enhanced PIT in drug-naïve rats can predict higher and more accurate rates of drug-self-administration.

The PIT paradigm has been used to understand the motivational influence of cues on instrumental performance, and it was demonstrated that alcohol addicted patients performed better in the PIT test than healthy controls (Garbusow et al., 2014; Schad et al., 2019). These two human studies suggest that a more pronounced PIT could be a marker for addictive behavior. In our study, however, contrary to these human findings, the transfer of the Pavlovian to instrumental response was not different between addicted-like and non-addicted-like rats. It has been previously demonstrated that prior cocaine experience may potentiate PIT (Saddoris et al., 2011), suggesting that enhanced performance during the PIT test in addicted patients may be caused by more frequent drug use than that of healthy controls. We could not confirm this finding since in this study cocaine addicted-rats have higher number of cocaine-associated responses and earn more cocaine infusions during CSA training.

Higher cocaine intake in addicted-like rats may have caused a stronger impairment of executive control, which could have led to more pronounced compulsive behavior and impacted instrumental learning and PIT (Jentsch and Taylor, 1999; Jentsch et al., 2002). However, analysis of the acquisition of instrumental learning in the PIT paradigm showed that addicted- and non-addicted-like animal groups were similar in their performance, ruling out the possibility of different learning capabilities in these rats. Nevertheless, to confirm that prior drug use did not affect animal performance during PIT, a group of drug naïve rats were tested in the PIT paradigm and then subjected to CSA. Similarly to the first experiment, a clear correlation was found between PIT and CSA. These results confirmed that PIT may be used to predict animal performance during drug cue-conditioned self-administration in rats.

Our experiments suggest that PIT responses do not correlate with addictive behavior but correlate with the extent of drug self-administration. Thus, the extent to which rats self-administer drugs (in the present study cocaine) and learn cue-drug associations is positively related to how strongly their instrumental behavior is under the influence of conditioned stimuli. The effect that the Pavlovian cue exerts on instrumental responding might be attributable to a general enhancement of motivation and/or to triggering an expectation of the instrumental outcome. In this respect, it is important to note that PIT effects correlate with the expectation that stimuli play a discriminative stimulus role in signaling the effectiveness of the instrumental response (Hogarth et al., 2014; Seabrooke et al., 2016; Hardy et al., 2017). Thus, it is possible that the correlation seen in our study between PIT responses and drug self-administration behavior reflect individual differences in awareness/attention to the discriminative function of stimuli. In conclusion, our data confirm that PIT can be used to predict individual performance in a drug self-administration paradigm but PIT alone is insufficient to predict or to be used as a marker of severity of substance use disorder. Furthermore, since two recent human studies indicate that PIT effects can predict relapse behavior in addicted patients (Garbusow et al., 2016; Sommer et al., 2018) further studies are needed to measure PIT effects and subsequent relapse behavior in the 0/3 crit model.

## Ethics Statement

All experimental procedures were approved by the Committee on Animal Care and Use (Regierungspräsidium Karlsruhe) and carried out in accordance with the local Animal Welfare Act and the European Communities Council Directive of 22 September 2010 (2010/63/EU).

## Author Contributions

TT contributed to designing experiments and analyzing the data, performed CSA and PIT experiments, and wrote the first draft of the manuscript. VV contributed to designing experiments, analyzing the data and writing the manuscript. TE established the PIT protocol. SR performed the experiment 2. RS contributed to designing experiments and writing the manuscript.

## Conflict of Interest Statement

The authors declare that the research was conducted in the absence of any commercial or financial relationships that could be construed as a potential conflict of interest.
